# Outbreak caused by the SARS-CoV-2 Omicron variant in the psychiatric ward of a general hospital in Brazil

**DOI:** 10.1590/0037-8682-0177-2022

**Published:** 2022-08-05

**Authors:** Tazio Vanni, Moema Silva Menezes, Letícia Olivier Sudbrack, Fabiana Futiwaki, Linda Stéphany Bezerra, Sergio Cabral, Edinan Oliveira, Paulo Giovanni Pinheiro Cortez, Fabiano José Queiroz Costa, Lucas Luiz Vieira, Mariana Matos Roll, Wildo Navegantes de Araújo, Maria Almiron, André Machado de Siqueira, Liliana Moscoso Ribeiro, Julival Fagundes Ribeiro

**Affiliations:** 1 Hospital de Base do Distrito Federal, Núcleo de Controle de Infecção Hospitalar, Brasília, DF, Brasil.; 2 Organização Pan-Americana da Saúde, Departamento de Emergência em Saúde, Brasília, DF, Brasil.; 3 Hospital de Base do Distrito Federal, Núcleo de Vigilância Epidemiológica, Brasília, DF, Brasil.; 4 Hospital de Base do Distrito Federal, Serviço de Psiquiatria, Brasília, DF, Brasil.; 5 Laboratório Central de Saúde Pública do Distrito Federal, Secretaria de Estado de Saúde do Distrito Federal, Subsecretaria de Vigilância em Saúde, Brasília, DF, Brasil.; 6 Universidade de Brasília, Faculdade UnB Ceilândia, Brasília, DF, Brasil.; 7 Fundação Oswaldo Cruz, Instituto Nacional de Infectologia Evandro Chagas, Brasília, DF, Brasil.

**Keywords:** COVID-19, SARS-CoV-2, Epidemiology, Surveillance, Infection control, Psychiatry

## Abstract

**Background:**

An outbreak of severe acute respiratory syndrome coronavirus 2 (SARS-CoV-2) Omicron variant was detected in the psychiatric ward of a general hospital in Brasília, Brazil.

**Methods:**

We report the investigation, clinical outcomes, viral sequencing, and control measures applied to outbreak containment.

**Results:**

The overall attack rate was 95% (23/24) in a period of 13 days. Among the cases, 78% (18/23) were vaccinated and 17% (4/23) required intensive care. The Omicron variant was isolated from the 19 sequenced samples.

**Conclusions:**

The findings highlight the potential harm that highly transmissible variants may generate among hospitalized populations, particularly those with comorbidities.

The increased transmissibility and potential immune evasion of new variants, such as the Omicron variant of concern (VOC), pose additional challenges to surveillance and infection control efforts in different settings^1^ ^-^ ^3^. Rapid detection and control of healthcare-associated outbreaks are paramount to prevent a wider spread of infection, cases, and deaths^4^ ^-^ ^6^. However, these measures are difficult to implement in settings such as psychiatric wards due to difficulties in risk awareness and clinical symptom communication^4^ ^,^ ^6^. 

Here, we report the investigation and response to an outbreak of severe acute respiratory syndrome 2 (SARS-CoV-2) Omicron VOC in the 24-bed closed psychiatric ward of a general hospital situated in Brasília, the capital of Brazil. In addition, we report the clinical outcomes, viral sequencing, and control measures applied for outbreak containment. The study was approved by the ethics committee of Instituto de Gestão Estratégica da Saúde do Distrito Federal (CAAE 56160322.0.0000.8153) and was conducted in accordance with the Declaration of Helsinki and International Council for Harmonization of Technical Requirements for Pharmaceuticals for Human Use (ICH) guidelines^7^ ^,^ ^8^.

On January 12, 2022, the first patient presenting with respiratory symptoms was detected in room 3 (red circle in Figure 1). In the following days, two other psychiatric patients presenting with respiratory symptoms were cohorted, and their asymptomatic contacts were tested and isolated. Nasopharyngeal samples were collected from these patients and tested for SARS-CoV-2 by reverse transcription-polymerase chain reaction (RT-PCR). Additional respiratory virus testing was performed. Two patients had a positive SARS-COV-2 test, and the other had a positive influenza A test result. On January 17, 2022, two symptomatic nosocomial cases were identified according to the Brazilian Health Regulatory Agency (ANVISA) guidelines^9^.

**FIGURE 1:**
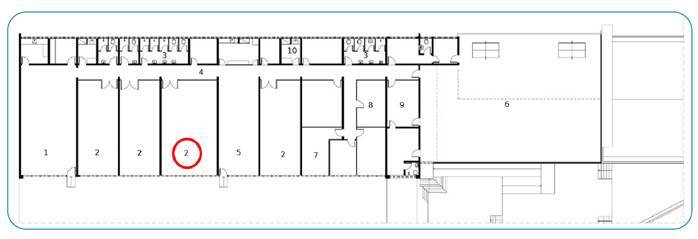
Basic floor plan - psychiatric ward. **Red circle:** location of probable index case, **1:** dining hall, **2:** bedrooms, **3:** bathrooms, **4:** corridor, **5:** living room, **6:** solarium, **7:** multipurpose space, **8:** physician’s office, **9:** meeting room, and **10:** medication room.

As soon as the first patients were identified, all parties involved in the assistance, surveillance, and infection control, as well as regional health authorities, were notified of a potential outbreak, and control measures were put in place. Control measures included: a) in-loco discussions of management protocols for patients presenting respiratory symptoms in both the ward and emergency unit; b) reinforcement of isolation and precaution measures, including cohorting of patients; c) strengthening of environmental decontamination and ventilation protocols; d) pause of new admissions and visitation; e) testing of all patients in the ward and emergency unit; f) testing of staff according to symptoms and exposure; g) daily monitoring of clinical status and laboratory data; and h) daily evaluation of conditions for discharge.

Despite these efforts, in a period of 13 days, among all 24 inpatients, 23 (overall attack rate was 95%) tested positive for SARS-CoV-2 (Figure 2). Twelve (52%) patients presented COVID-19 symptoms, as described in Table 1. Eight (35%) patients presented with severe acute respiratory illness (SARI), and four (17%) were admitted to the intensive care unit (ICU). None of the patients died or required mechanical ventilation. It is worth mentioning that five staff members reported coronavirus disease 2019 (COVID-19) symptoms and tested positive. However, the clinical and laboratory information of staff members was subjected to occupational medicine policies and could not be verified.

**FIGURE 2:**
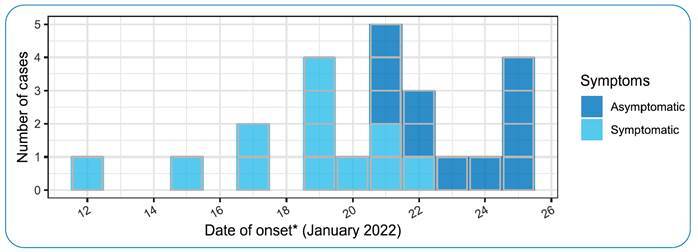
Distribution of cases in the psychiatry ward, January 12-February 8, 2022 (n = 23). *Date of symptom onset or date of positive test, if asymptomatic.

**TABLE 1:** Baseline and clinical characteristics of patients (n = 23).

Characteristics	Value	
**Age**	50 (mean)	36-65 (IQR)
Sex (male)	65.2%	15/23
Mental illness*		
Schizophrenia	26.1%	6/23
Bipolar disorder	34.8%	8/23
Alcohol use disorder	8.7%	2/23
Organic mental disorder	0.0%	0/23
Dementia	17.4%	4/23
Retardation	8.7%	2/23
Other mental illnesses	21.7%	5/23
Medical illness*		
Hypertension	34.8%	8/23
Diabetes	17.4%	4/23
Heart disease	4.3%	1/23
Chronic Renal Disease	0.0%	0/23
Chronic Liver Disease	0.0%	0/23
Chronic Lung Disease	8.7%	2/23
Other illnesses	47.8%	11/23
Symptoms		
Fever	39.1%	9/23
Chills	0.0%	0/23
Cough	39.1%	9/23
Diarrhea	13.0%	3/23
Abdominal pain	0.0%	0/23
Mialgia	4.3%	1/23
Fatigue	21.7%	5/23
Sore throat	8.7%	2/23
Headache	8.7%	2/23
Rhinorrea	8.7%	2/23
Smell disorder	0.0%	0/23
Taste disorder	0.0%	0/23
Dyspnea	4.3%	1/23
Thoraxpressure	4.3%	1/23

*Duplication allowed.

The mean age of the 23 patients was 50 years (interquartile range [IQR] 36-65), and the male-to-female ratio was 1.8:1. Bipolar disorder was the most common mental illness, accounting for 35% (8/23) of all cases. The most common underlying disease was hypertension, which accounted for approximately 35% (8/23) of patients. Cough, fever, and diarrhea were the most frequent symptoms reported by patients (Table 1). In addition, four patients admitted to the ICU presented radiologic signs of COVID-19-associated pneumonia.

Vaccination status could not be accurately assessed through patient interviews. According to the National Immunization Program (SI-PNI) database, 78% (18/23) of patients received at least one vaccine dose. In addition, 60% (14/23) of the patients received two or more doses. Only two patients received three doses. The mean time since the last vaccination was 147 days (IQR 75-193). Three vaccines of three different classes were administered to this population: inactivated virus (CoronaVac; Sinovac), viral vector (ChAdOx1 nCoV-19; AstraZeneca), and messenger ribonucleic acid (mRNA) (BNT162b2; Pfizer). It is worth mentioning that none of the four patients admitted to the ICU received a third vaccine dose (booster). Two received two doses of CoronaVac, one of which received two doses of BNT162b2, and the other received only one dose of BNT162b2.

Regarding testing and sequencing, the hospital protocol recommends that all patients should be tested for SARS-CoV-2 prior to admission, regardless of symptoms. Therefore, nasopharyngeal samples were collected from symptomatic patients and tested by RT-PCR, apart from one patient who was tested with a rapid antigen test. Asymptomatic patients were tested by RT-PCR. All RT-PCR SARS-CoV-2 positive samples with cycle threshold (Ct) values below 28 (n = 19; 82%) were sequenced and screened for SARS-CoV-2 variants in the Central Laboratory of Public Health (LACEN-DF). 

Total nucleic acids were extracted from nasopharyngeal swab specimens using the Quick-DNA/RNA™ Viral MagBead kit (Zymo Research). SARS-CoV-2 RNA was detected by multiplex real-time RT-PCR (rRT-PCR) using a BIOMOL OneStep/COVID-19 kit (IBMP). The samples were submitted for partial sequencing of the spike gene (S gene) and the genomic region between ORF8 and the nucleocapsid gene (N gene) using the Sanger method. Sequencing data analysis was performed using Geneious ™ software (version 11.1.5) with Geneious alignment, de novo assembly, and mapping to reference tools. The consensus sequences were mapped to the SARS-CoV-2 reference sequence (NC_045512.2) and compared with that of the most prevalent lineages available on the Global Initiative on Sharing All Influenza Data (GISAID). All the samples were classified as Omicron VOC. In six samples, the same single nucleotide substitution (from cytosine to thymine) was noticed at nt 21595, which corresponds to a synonymous mutation. 

Nosocomial outbreaks of SARS-CoV-2 have been reported among patients and healthcare workers in hospitals and long-term term care facilities^4^ ^-^ ^6^ ^,^ ^10^. However, due to the recent emergence of Omicron VOC, there is limited understanding of their epidemiological and clinical presentation in different settings. For example, in the psychiatric ward of a general hospital, we found high transmissibility of the Omicron VOC, with an overall attack rate of 95%.

A recent report of an outbreak related to Omicron in a party^3^ also reported a high attack rate (74%). However, the patterns of transmission were different because the individuals were exposed to an event in a crowded indoor location with a long exposure time. The onset dates coincided for most cases. In the psychiatric ward, transmission among patients probably took place over a longer period of time. Consequently, the onset dates of cases were distributed over a week. We cannot exclude the possibility of multiple virus introductions by visitors or staff, considering the same mutation found in six samples.

The clinical presentation of Omicron disease among psychiatric patients with comorbidities differs from that described in the general population^3^. Although upper respiratory tract symptoms were predominant, 17% (4/23) of the patients presented with pneumonia requiring oxygen therapy. These findings highlight the potential harm that Omicron may cause among hospitalized populations, particularly those with comorbidities, even among vaccinated individuals.

In light of the rapidly evolving variants, further studies are necessary to gain an understanding of the distinct variant dynamics in different populations, as well as the effectiveness of control measures, particularly among psychiatric inpatients.
